# mGem: Revisiting bacterial overflow metabolism

**DOI:** 10.1128/mbio.01193-25

**Published:** 2025-08-25

**Authors:** Niaz Bahar Chowdhury, Wheaton L. Schroeder, Lummy Monteiro, Kristin E. Burnum-Johnson

**Affiliations:** 1 The Environmental Molecular Sciences Laboratory, Pacific Northwest National Laboratory 6865, Richland, Washington, USA; 2 Voiland School of Chemical Engineering and Bioengineering, Washington State University 744660, Pullman, Washington, USA; 3 Biological Science Division, Pacific Northwest National Laboratory 6865, Richland, Washington, USA; Instituto Carlos Chagas, Curitiba, Brazil

**Keywords:** overflow metabolism, bioenergetics, proteome allocation, thermodynamic

## Abstract

Bacterial overflow metabolism, where cells perform oxidative fermentation despite the availability of ample oxygen and carbon sources, remains a long-standing paradox in microbial metabolism. Traditional explanations attribute this phenomenon to bacterial physiology, including rapid growth, redox imbalances, competitive advantages in microbiomes, and catabolite repression. However, recent advances in systems biology have revealed additional contributing factors, such as thermodynamic constraints, proteome allocation efficiency, bioenergetics, and the membrane real estate hypothesis. Despite these insights, a comprehensive commentary that critically examines these perspectives is still lacking. In this mGem, we summarize key drivers of overflow metabolism, examine state-of-the-art theories, and identify unresolved questions in current understanding. By evaluating multiple viewpoints, we aim to provide a cohesive analysis of bacterial overflow metabolism and contribute to a broader understanding of microbial physiology, regulatory networks, and evolutionary adaptations shaping metabolic strategies.

## PERSPECTIVE

**Fig 1:**
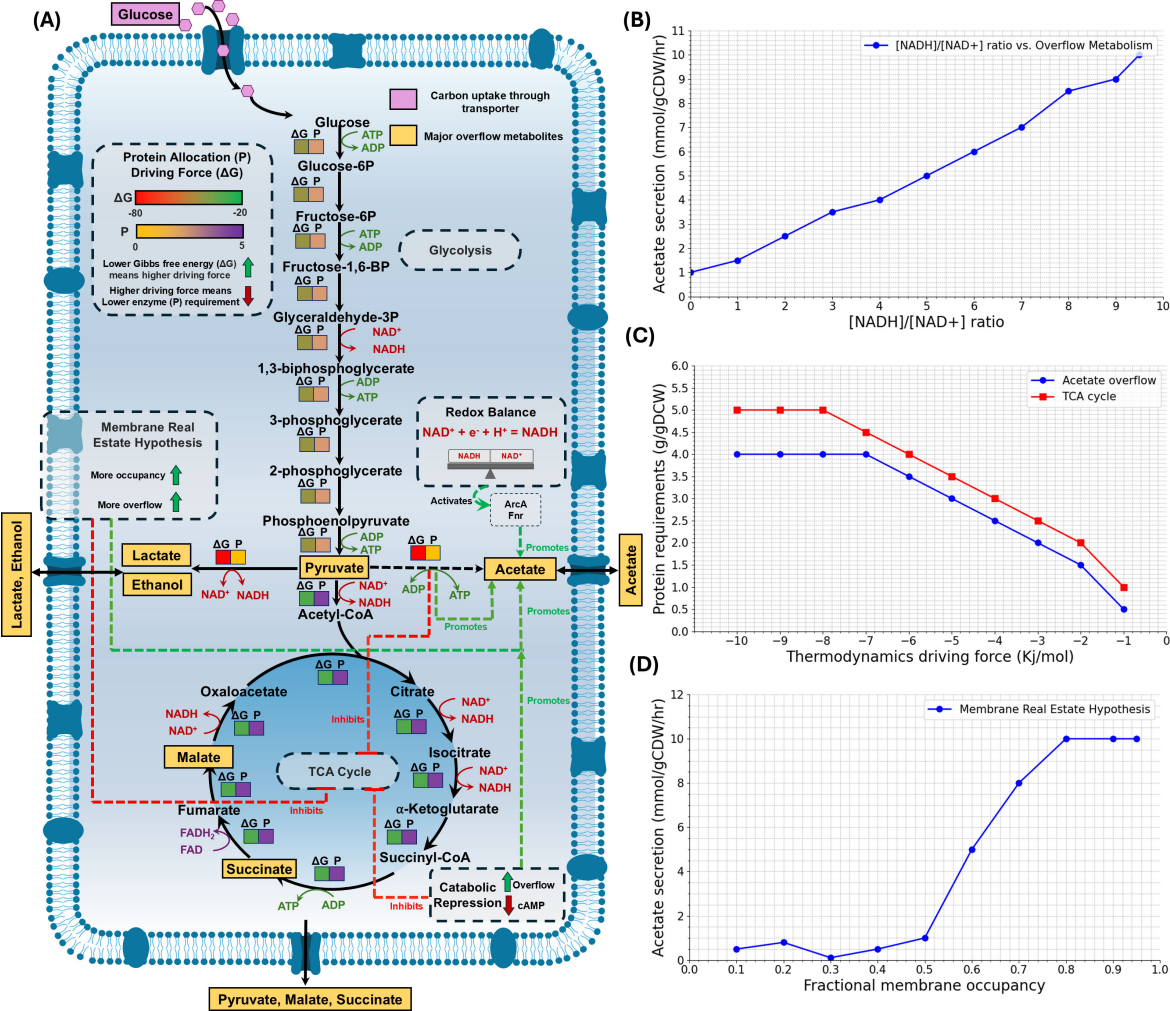
Overview of known mechanisms driving overflow metabolism. (**A**) Key hypotheses explaining overflow metabolism include carbon catabolite repression, redox imbalances, protein allocation linked to thermodynamic driving force, and the membrane real estate hypothesis. (**B**) Acetate secretion decreases as reducing power availability increases, emphasizing its relationship with redox balance. (**C**) Acetate overflow pathways exhibit higher thermodynamic driving forces, allowing for lower protein requirements and efficient ATP production. (**D**) Increased membrane occupancy correlates with higher acetate secretion, suggesting that limited transport capacity promotes overflow. Note: data in panels B–D represent hypothetical trends, not experimental results.

Overflow metabolism, while a hallmark of fast-growing microorganisms such as bacteria and yeast, is not restricted to these domains of life. Notably, similar metabolic phenomena have been observed in mammalian systems, including cancer cells (the Warburg effect) and certain immune cells. These systems also display the preference for less energy-efficient pathways even in the presence of oxygen and ample nutrients, as described in classic and recent works (1–3). This behavior perplexes researchers as it is less efficient than oxidative phosphorylation in terms of ATP yield. Historically, explanations for this phenomenon have focused on factors like redox imbalances, competitive advantages in microbiomes, and carbon catabolite repression (CCR), which influence metabolic priorities such as rapid growth rate and lead to byproduct excretion and enhanced selective advantage (4, 5). However, recent advances in systems biology have offered fresh insights, considering factors such as thermodynamic constraints and proteome allocation strategies, limited inner membrane space, and the membrane real estate hypothesis (4, 5) (Fig. 1A). These new perspectives add complexity to our understanding of overflow metabolism, highlighting that metabolism is more than raw efficiency and ATP yield. Given the new focus on overflow metabolism, it is essential to synthesize existing knowledge, both existing and emerging, by evaluating past and present theories; their contradictions and synergies; and highlighting gaps in research. A comprehensive understanding of overflow metabolism has significant practical implications, particularly for the optimization of microbial cell factories. Addressing overflow metabolism could lead to tangible improvements in industrial biotechnology by enhancing microbial productivity, pH tolerance, redox balance, and overall process efficiency. Therefore, this effort seeks to form a cohesive understanding of bacterial overflow metabolism and its implications for microbial metabolism.

## CURRENT UNDERSTANDINGS

### Fueling fast growth: how carbon source preference shapes bacterial metabolism

Overflow metabolism can result from an imbalance in intracellular carbon flux. When microorganisms (such as *Escherichia coli* or *Bacillus subtilis*) find themselves in glucose-rich environments, their glycolytic activity tends to increase significantly to meet the higher ATP demands due to elevated growth rates (6). This heightened glycolytic pathway activity results in an overproduction of acetyl-CoA, a central metabolic intermediate between glycolysis, fatty acid metabolism, the tricarboxylic acid (TCA) cycle, and some amino acid metabolisms.

The TCA cycle is responsible for oxidizing acetyl-CoA to produce ATP and reduce electron carriers (primarily NAD^+^ and FAD^+^), yet has limited capacity (7). When acetyl-CoA production surpasses the TCA cycle’s ability to process it, the excess is generally converted to some fermentation product, with the product or products produced varying by species. These include lactate (to oxidize NADH), ethanol (to oxidize NAD(P)H), malate (to oxidize NADH), acetate (allowing for additional ATP generation through substrate-level phosphorylation), pyruvate (maintaining redox balance), formate (from pyruvate formate lyase, avoiding NAD^+^ reduction in glycolysis), succinate (oxidizing NADH), and hydrogen gas (to oxidize NAD(P)H, ferredoxin, and ferrocytochrome), among other products. However, these are generally considered “waste” products (particularly in carbon-limited growth conditions) in microbial cell factories, diverting carbon flux away from desirable products (8, 9) leading to interest in overflow metabolism, particularly avoidance or tight regulation (10, 11). For instance, *E. coli* achieves higher growth rate by employing overflow metabolism, where the surplus acetyl-CoA is diverted to acetate production to produce ATP, supporting its enhanced metabolic activities (12). *B. subtilis* (13) also exhibits similar strategies, secreting acetate to cope with the high glycolytic throughput during rapid growth phases induced by nutrient abundance.

The complexity of overflow metabolism is further compounded by CCR, a sophisticated regulatory mechanism that optimizes energy production by prioritizing glycolysis over other metabolic pathways (14). CCR operates via a regulatory network involving the cyclic AMP (cAMP) and cAMP receptor protein (CRP) complex. In glucose-rich conditions, cAMP levels drop, leading to reduced activation of CRP-dependent genes, many of which are integral to the TCA cycle and secondary carbon-source utilization (15). This regulatory strategy ensures that cells can efficiently exploit a glucose-rich environment by channeling carbon primarily through glycolysis, which results in rapid ATP production and abundant supplies of metabolic precursors necessary for biomass synthesis. By effectively suppressing less efficient or unnecessary metabolic pathways, CCR enables cells to maximize energy extraction from available glucose, supporting fast proliferation. Furthermore, this prioritization of glycolysis provides a robust mechanism to maintain cellular homeostasis, as cells can swiftly adjust their metabolic output in response to fluctuations in external nutrient availability, thus optimizing survival and growth in dynamic environments.

### Balancing cellular energy: how redox state influences bacterial metabolism

Next, redox balance within the cell, represented by the NADH/NAD^+^ ratio, is a key factor governing overflow metabolism (Fig. 1B) (16–18). The most straightforward argument for this is that the TCA cycle reduces electron carriers; whereas, in contrast, overflow metabolism avoids electron carrier reduction (in producing pyruvate and formate) or oxidizes these carriers (in producing lactate, ethanol, malate, and hydrogen gas) and effectively carries excess electrons out of the cell as these products are exported. Therefore, the ratio of TCA cycle to overflow metabolism flux is potentially a significant mechanism of control for the redox state of a cell. This is emphasized in recent investigations on the role of the NADH/NAD^+^ ratio in modulating overflow metabolism (17). By expressing a water-forming NADH oxidase from *Streptococcus pneumoniae*, researchers were able to manipulate the redox ratio in *E. coli* and establish a strong correlation between acetate formation and the NADH/NAD^+^ ratio using steady-state chemostat cultures (16).

The reduction in the NADH/NAD^+^ ratio, through expression of NADH oxidase, was shown to alleviate the repression of several genes associated with the TCA cycle and respiration, which typically occurs as glucose consumption increases (19). Genome-wide transcription analyzes revealed that the expression of NADH oxidase counteracted this repression, suggesting an intricate relationship between the redox ratio and metabolic regulation. Further analysis of promoter binding sites upstream of genes correlated with this redox ratio unearthed a degenerate sequence strongly resembling ArcA binding sites. ArcA, a redox-sensitive regulatory protein, plays a central role in this process. The deletion of this *arcA* homolog resulted in reduced acetate production and increased biomass yield, attributed to enhanced TCA cycle and respiratory pathway capacities. Crucially, acetate formation was eliminated by reducing the redox ratio through NADH oxidase expression in the *arcA* mutant, even under very high glucose consumption scenarios (19).

In many bacteria, redox-sensing regulatory proteins are central in adapting metabolic processes in response to environmental and intracellular changes in oxygen and redox status. For example, in *E. coli*, ArcA and Fnr are two well-studied transcriptional regulators that respond to such cues (20). ArcA is typically activated under low oxygen conditions by increased NADH accumulation and acts by repressing genes linked with aerobic respiration, thereby encouraging shifts toward fermentative or overflow pathways that efficiently accommodate excess reducing equivalents. Fnr, on the other hand, is activated in anaerobic environments and promotes the expression of genes beneficial for anaerobic growth, further steering metabolism toward overflow or fermentative pathways. It should be noted that not all bacteria possess ArcA or FNR homologs; different species utilize distinct regulatory strategies to sense and respond to redox changes. In general, these redox-responsive regulators exemplify how bacteria refine their metabolic adaptation based on redox status and environmental cues, enhancing survival and growth and providing a foundation for further discovery of regulatory mechanisms prevalent at reduced NADH/NAD^+^ ratios.

### Making the most of resources: how protein allocation and energy efficiency drive overflow metabolism

Proteome allocation involves the distribution of cellular resources to synthesize proteins that manage various metabolic pathways. Under conditions that demand rapid growth, cellular systems may prioritize pathways like acetate overflow metabolism (Fig. 1C), which require fewer proteins to generate quick ATP compared to the more protein-intensive TCA cycle (12, 21–23). This allocation strategy can favor overflow metabolism because it enables rapid energy production without the necessity of fully oxidizing glucose to CO_2_ through the TCA cycle. The allocation strategy showcases the cellular economy in protein usage to maximize growth under nutrient-abundant conditions.

The thermodynamic efficiency of TCA cycle and PTA-ACKA pathways also influences overflow metabolism. The partial oxidation of glucose to acetate may be more thermodynamically favorable under some circumstances, conserving ATP that would otherwise be used in more complex metabolic pathways (24). The production of acetate allows cells to bypass ATP-consuming steps of complete oxidation, conserving energy for fast-growing and division-related processes. These considerations illustrate the strategic metabolic decisions cells make to balance energy production with growth and reproduction. Very recently, proteome allocation and bacterial cell heterogeneity have been combined to propose an extension of the proteome allocation hypothesis, which predicts overflow metabolism well for *E. coli* (25). This extension adds an additional layer by accounting for heterogeneity within bacterial populations and incorporating the idea that overflow metabolism may confer a competitive advantage under certain environmental conditions, such as nutrient fluctuations or competition within microbial communities.

### Space matters: how limited membrane area shapes bacterial metabolism

An additional layer to the understanding of overflow metabolism is provided by the membrane real estate hypothesis (26–28). The membrane real estate hypothesis (Fig. 1D) provides an interesting layer to understanding overflow metabolism by focusing on the spatial constraints within bacterial cells, particularly relating to the inner membrane where respiratory pathway enzymes are housed. This hypothesis, driven by computational modeling, suggests that cells, such as *E. coli*, face limitations due to the finite space available on the membrane, which hosts complex proteins essential for processes like the electron transport chain (29). Given this spatial restriction, bacterial cells may be inclined to prioritize metabolic pathways that demand less involvement of the membrane, such as glycolysis.

Unlike respiration, overflow metabolism is conducted in the cytosol and does not require membrane-bound complexes, allowing cells to produce ATP without being bottlenecked by membrane occupancy limits. In nutrient-rich conditions, where rapid energy production is critical, catabolizing more glucose helps cells process large amounts of glucose quickly, even if this comes at the expense of energy yield. This preference inherently leads to overflow metabolism, as high glycolytic flux generates more pyruvate than can be handled by the membrane-limited constrained TCA cycle and electron transport chain, resulting in the conversion of excess pyruvate to overflow metabolites like acetate, lactate, and ethanol (12, 21–23).

The implications of the membrane real estate hypothesis are significant in that they highlight how physical constraints—the limited capacity of cellular membranes—can dictate metabolic strategies. Bacterial cells adapt not only to metabolic demands but also to spatial limitations, leveraging pathways that circumvent heavy reliance on membrane space. This perspective enhances the understanding of overflow metabolism’s persistence, illustrating it as an adaptation aligned with cellular architecture constraints and energy demands in diverse environments.

## GAPS IN UNDERSTANDING AND URGENT NEEDS

Understanding overflow metabolism remains a challenging endeavor due to the absence of a mechanistic model that integrates various known factors, including carbon flux imbalances, redox states, proteome allocation, and membrane constraints. While these elements have been extensively studied, they are often addressed in isolation. Therefore, there is an urgent need to develop a comprehensive model that captures the interplay of these factors, enabling the prediction of cellular behavior across different conditions.

Recent literature offers promising developments in this area. Shahreen et al. (24) have successfully integrated thermodynamic driving forces and the membrane real estate hypothesis with genome-scale metabolic models to improve acetate overflow predictions in *Staphylococcus aureus*. This advancement suggests that incorporating additional known factors could yield a more hierarchical understanding of overflow metabolism. Furthermore, resource allocation-based models such as metabolic and expression models (30, 31), resource balance analysis models (32), and whole-cell models (33) provide valuable frameworks for developing a hierarchical understanding of the various elements of overflow metabolism.

To address the remaining gaps, future studies could leverage specific experimental approaches, such as dynamic chemostat or continuous culture experiments and high-resolution proteomics to directly quantify enzyme and resource allocation under differing metabolic states.

Finally, exploring multi-omics data (transcriptomics, proteomics, metabolomics, lipidomics, etc.) is relevant to overflow metabolism and integrating it into systems biology models offers organism-specific insights (e.g., acetate secretion in *E. coli* [12]) and fermentation product-specific differences (such as acetate versus lactate production depending on the species and pathway regulation [34]). These approaches can help uncover both unique and shared features of overflow metabolism across taxa. A more structured roadmap for the future could involve stepwise integration of modeling layers—starting from core metabolic models, then adding gene expression constraints (e.g., ME-models), and ultimately incorporating spatial or structural factors such as membrane occupancy and cell architecture. Experimentally testing predictions at each stage would help refine and validate these frameworks. AI-based agentic workflows, combined with large-scale automation capabilities, therefore seem critical along the path to predictive understanding (35). Finally, combining both systems biology models and multi-modal ML-derived regulatory network can give us the much-desired hierarchical understanding of overflow metabolism.
